# Diagnostic Challenge in *PLIN1*-Associated Familial Partial Lipodystrophy

**DOI:** 10.1210/jc.2019-00849

**Published:** 2019-06-27

**Authors:** Isabelle Jéru, Marie-Christine Vantyghem, Elise Bismuth, Pascale Cervera, Sara Barraud, Martine Auclair, Camille Vatier, Olivier Lascols, David B Savage, Corinne Vigouroux, PLIN1-Study Group

**Affiliations:** 1 Sorbonne University, Inserm U938, Saint-Antoine Research Centre and Institute of CardioMetabolism and Nutrition, Paris, France; 2 Department of Molecular Biology and Genetics, Assistance Publique-Hôpitaux de Paris, Saint-Antoine University Hospital, Paris, France; 3 Department of Endocrinology, Diabetology and Metabolism, Lille University Hospital, Lille, France; 4 Inserm U1190, European Genomic Institute for Diabetes, Lille, France; 5 Department of Pediatric Endocrinology and Diabetology, Competence Center for Rare Diseases of Insulin Secretion and Insulin Sensitivity, Paris-Diderot University, Sorbonne Paris Cité, Assistance Publique-Hôpitaux de Paris, Robert Debré University Hospital, Paris, France; 6 Department of Pathology, Sorbonne University, Inserm U938, Assistance Publique-Hôpitaux de Paris, Saint-Antoine University Hospital, Paris, France; 7 Department of Endocrinology, Diabetes, Nutrition, Reims University Hospital, Reims, France; 8 Department of Endocrinology, Diabetology and Reproductive Endocrinology, National Reference Center for Rare Diseases of Insulin Secretion and Insulin Sensitivity, Assistance Publique Hopitaux de Paris, Saint Antoine University Hospital, Paris, France; 9 Metabolic Research Libraries, University of Cambridge, Wellcome Trust-MRC Institute of Metabolic Science, Cambridge, United Kingdom

## Abstract

**Context:**

Heterozygous frameshift variants in *PLIN1* encoding perilipin-1, a key protein for lipid droplet formation and triglyceride metabolism, have been implicated in familial partial lipodystrophy type 4 (FPLD4), a rare entity with only six families reported worldwide. The pathogenicity of other *PLIN1* null variants identified in patients with diabetes and/or hyperinsulinemia was recently questioned because of the absence of lipodystrophy in these individuals and the elevated frequency of *PLIN1* null variants in the general population.

**Objectives:**

To reevaluate the pathogenicity of *PLIN1* frameshift variants owing to new data obtained in the largest series of patients with FPLD4.

**Methods:**

We performed histological and molecular studies for patients referred to our French National Reference Center for Rare Diseases of Insulin Secretion and Insulin Sensitivity for lipodystrophy and/or insulin resistance and carrying *PLIN1* frameshift variants.

**Results:**

We identified two heterozygous *PLIN1* frameshift variants segregating with the phenotype in nine patients from four unrelated families. The FPLD4 stereotypical signs included postpubertal partial lipoatrophy of variable severity, muscular hypertrophy, acromegaloid features, polycystic ovary syndrome and/or hirsutism, metabolic complications (*e.g.,* hypertriglyceridemia, liver steatosis, insulin resistance, diabetes), and disorganized subcutaneous fat lobules with fibrosis and macrophage infiltration.

**Conclusions:**

These data suggest that some FPLD4-associated *PLIN1* variants are deleterious. Thus, the evidence for the pathogenicity of each variant ought to be carefully considered before genetic counseling, especially given the importance of an early diagnosis for optimal disease management. Thus, we recommend detailed familial investigation, adipose tissue-focused examination, and follow-up of metabolic evolution.

## Teaser

We have described novel heterozygous PLIN1 frameshift variants in the largest cohort of FPLD4 reported to date and provide a set of arguments for their pathogenicity.

Familial partial lipodystrophy (FPLD) syndromes are rare diseases characterized by a limited capacity of peripheral fat to store triglycerides, which results in metabolic abnormalities including insulin resistance, hypertriglyceridemia, liver steatosis, and polycystic ovary syndrome (1). Heterozygous frameshift variants in the *PLIN1* gene encoding perilipin-1 have been identified in six families (2–4), thereby defining the FPLD type 4 (FPLD4) subtype.

Perilipin-1 is a structural lipid droplet protein that facilitates triglyceride storage or initiates lipolysis, depending on the hormonal stimuli. We have shown that several FPLD4-associated *PLIN1* frameshift variants disrupt the ability of perilipin-1 to inhibit basal lipolysis in adipocytes (2, 3, 5). However, the pathogenicity of other heterozygous *PLIN1* null variants, identified in patients referred for evaluation of maturity onset diabetes of the young, hyperinsulinemic hypoglycemia, or type 2 diabetes, has recently been questioned by Laver *et al.* (6).

Since proper interpretation of *PLIN1* variants is crucial for genetic counseling, the aim of the present study was to determine the pathogenicity of *PLIN1* null variants in the light of the new genotype–phenotype data obtained in the largest series of patients with FPLD4 reported to date.

## Subjects and Methods

Sequencing of a panel of lipodystrophy genes (*AGPAT2, AKT2, BSCL2, CAV1, CIDEC, LIPE, LMNA, PLIN1, POLD1, PPARG, PTRF,* and *ZMPSTE24*) was performed in 237 independent index cases, investigated in our French National Reference Network for Rare Diseases of Insulin Secretion and Insulin Sensitivity, for manifestations evocative of a lipodystrophic syndrome. Capture (SeqCap EZ enrichment protocol, Roche NimbleGen, Roche Sequencing, Pleasanton, CA) was followed by massively parallel sequencing on a MiSeq platform (Illumina Inc., San Diego, CA). The data were analyzed using the Sophia Genetics DDM pipeline^®^. *PLIN1* frameshift variants were confirmed by Sanger sequencing. Affected relatives were identified after familial investigations. Abdominal subcutaneous adipose tissue biopsy specimens were obtained from two patients. Western blot and histological analyses were performed as previously described (2). All the subjects provided written informed consent in accordance with the legal procedures for molecular and histological investigations and publication of photographs. The Comité de Protection des Personnes Ile-de-France 5 (Paris, France) approved the present study.

## Results

### Molecular diagnosis

**Figure 1.**
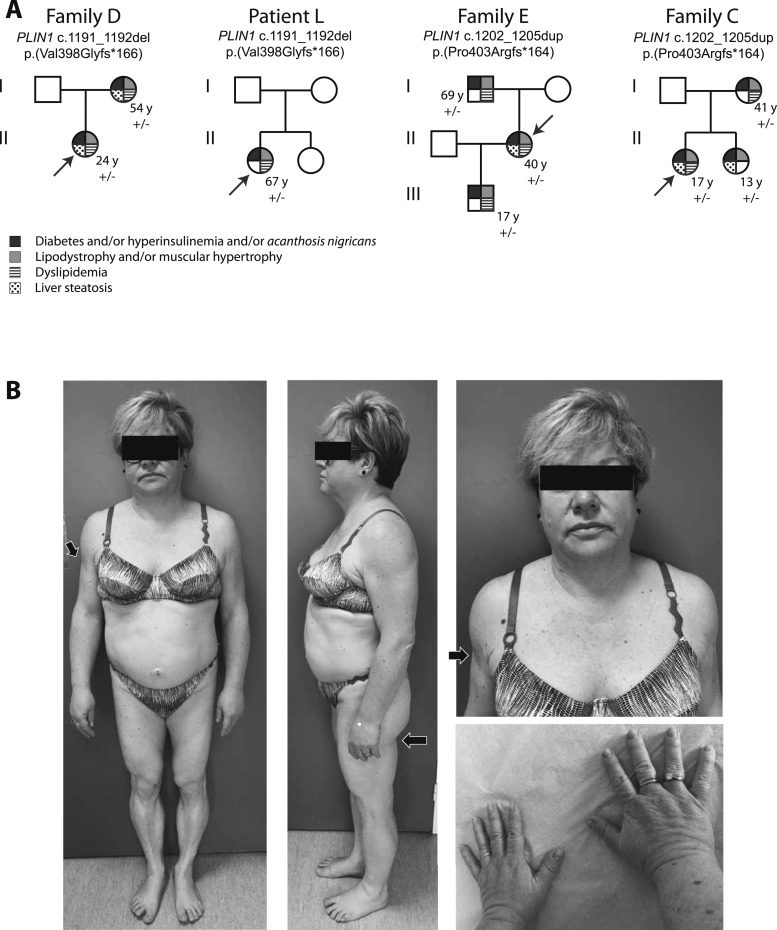
(A) Molecular and clinical investigations of patients with heterozygous *PLIN1* null variants. *Arrows* indicate index cases. Genealogical trees show a cosegregation of *PLIN1* variants with the disease phenotype. The nomenclature of *PLIN1* variants is based on the RefSeq accession numbers NM_002666.5 and NP_002657.3. (B) Morphotype of patient E-II2 with partial lipodystrophy and acromegaloid features. Subcutaneous lipoatrophy of the upper and lower limbs can be observed, with muscle hypertrophy (*arrows*). Compared with patients with FPLD type 2 (*LMNA*-linked FPLD), the neck is less broad, and the breast and subcutaneous abdominal fat are not affected by lipoatrophy. The acromegaloid features include face infiltration, a slightly enlarged nose, deep wrinkles, thick lips and hands, and enlarged feet.

**Figure 2.**
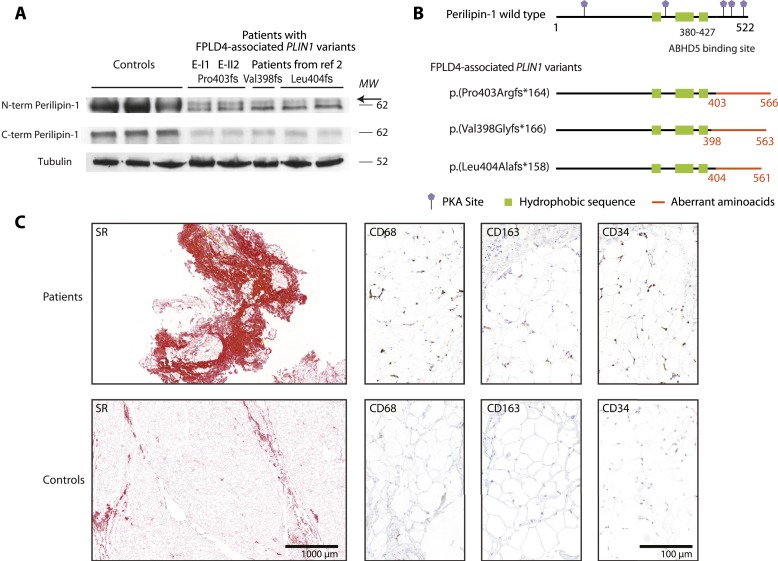
Study of subcutaneous adipose tissue from patients. (A) Perilipin-1 expression in abdominal subcutaneous adipose tissue from patients E-II2 and E-I1 compared with controls and previously described patients with FPLD4 (2). Western blot of whole cell extracts was performed using antibodies directed against the N-terminal and C-terminal parts of wild-type perilipin-1, as previously described (2). The mutant isoform was recognized by the N-terminal antibody as an additional band (*arrow*) just above the 62-kD molecular weight (MW) marker, which was not detected by the C-terminal antibody. Tubulin (antibody T5168, Sigma-Aldrich, St. Quentin-Fallavier, France) was used as a loading control. (B) Consequences on perilipin-1 protein expression of FPLD4-associated *PLIN1* frameshift variants studied in (A). (C) Histological and immunohistological analyses of abdominal subcutaneous adipose tissue from patients E-I1 and E-II-2 compared with controls. Adipose tissue samples from patients displayed disorganized fat lobules of heterogeneous size, with increased fibrosis [Sirius red (SR) staining, 16% to 35% of the total sample surface vs 0.2% to 3% in controls], increased vascularization (density of CD34 staining, 0.13% to 0.44% in adipose lobules from patients vs 0.001% to 0.004% in controls), and increased macrophage infiltration with crown-like structures (assessed by CD163 and CD68 staining, density per 10^−5^ μm^2^: 1.9 to 2.2 and 3.6 to 4.3 in patients vs 0.0 and 0.0 to 0.01 in controls, respectively).

A heterozygous *PLIN1* frameshift variant was identified in four index cases and five affected relatives (Fig. 1A). Patients from family D and L carried the c.1191_1192del deletion, previously shown to be expressed as an abnormal p.(Val398Glyfs*166) elongated form of perilipin-1, leading to constitutive activation of basal lipolysis (2, 5). In family E and C, we identified a 4bp-duplication in exon 8 (c.1202_1205dup), leading to the synthesis of a p.(Pro403Argfs*164) mutant protein, whose expression in adipose tissue was confirmed by Western blot (Fig. 2). These variants were not found in public databases (Exome Aggregation Consortium, Genome Aggregation Database) and cosegregated with the disease within each family (Fig. 1A). In all patients, we did not find any other molecular defect in known lipodystrophy genes.

### Disease phenotype

The clinical and biological features of the nine investigated family members carrying a *PLIN1* variant recapitulated the FPLD4 cardinal signs (2) (*i.e.,* lipoatrophy, muscular hypertrophy, facial acromegaloid features, insulin resistance-related ovarian dysfunction, and metabolic complications (*e.g.,* hyperinsulinemia or insulin-resistant diabetes, hypertriglyceridemia, liver steatosis) (Table 1). The index cases had initially been referred for ovarian hyperandrogenism with lipodystrophy (patient D-II3), suspicion of acromegaly (patients L and E-II2), or early-onset nonautoimmune diabetes (patient C-II1). In several individuals (patients D-I2, C-I2, C-II2), the disease manifestations were only detected after family studies.

**Table 1.** Characteristics of Patients Investigated in the Present Study

Characteristic	Family D		Family L	Family E			Family C			Mean ± SD	Median (Range)
Patient number	D-I2	D-II3*	L*	E-I1	E-II2*	E-III1	C-I2	C-II1*	C-II2	NA	NA
Sex	Female	Female	Female	Male	Female	Male	Female	Female	Female	NA	NA
Age, y	54	24	67	69	40	17	41	17	13	38 ± 21.7	40 (13–69)
BMI, kg/m^2^	23	25.5	22.7	25.7	30.7	23.8	29.8	25.4	19.6	25.1 ± 3.5	25.4 (19.6–30.7)
Origin	France	France	France	France	France	France	France	France	France		
Age at diagnosis, y	54	15	40	58	38	17	41	15	13	32.3 ± 17.7	38 (13–58)
Lipodystrophy	Android habitus; four limb lipoatrophy	Android habitus; four limb lipoatrophy	Generalized lipoatrophy	Four limb, gluteal and trunk lipoatrophy; cervicofacial fat accumulation	Lower limb and gluteal lipoatrophy	Mild lower limb, gluteal and trunk lipoatrophy	Android habitus; four limb and gluteal lipoatrophy; cervicofacial fat accumulation	Android habitus; lower limb and gluteal lipoatrophy; cervicofacial fat accumulation	Mild lower limb lipoatrophy	NA	NA
Acromegaloid features	Present	Present	Present	Absent	Present	Present	Present	Present	Absent	NA	NA
Muscular hypertrophy	Present	Present	Present	Present	Present	Present	Present	Present	Not obvious	NA	NA
Acanthosis nigricans	Absent	Present	Absent	Absent	Present	Absent	Present	Present	Absent	NA	NA
Total fat (DEXA), %	23.1	NA	12	16.4	21	13	NA	NA	NA	17.1 ± 4.9	16.4 (12–23.1)
Serum leptin, ng/mL	1.7	1.1	1.9	6.5	7.0	3.2	NA	12	NA	4.8 ± 3.9	3.2 (1.1–12)
Glucose tolerance	Gestational diabetes at age 20, 23, and 30 y; permanent diabetes at age 34 y	Impaired glucose tolerance at age 15 y; diabetes at age 20 y	Diabetes diagnosed at age 40 y	Diabetes diagnosed at age 58 y	Gestational diabetes at age 30 y; permanent diabetes at age 38 y	Normal glucose tolerance with increased fasting insulin	Impaired fasting glucose; antecedent of gestational diabetes	Diabetes diagnosed at age 15 y	Normal fasting glucose with increased fasting insulin	NA	NA
Serum fasting glucose/insulin, mmol/L/pmol/L	8/NA (insulin therapy); preserved C-peptide (1.2 nmol/L)	6.1/402 at age 15 y with BMI 24 kg/m^2^	3.9/NA (insulin therapy)	8.3/NA	7.4/112	4.5/65.2	6.1/456	7.2/NA (insulin therapy); high C-peptide (3.3 nmol/L)	4.6/257	6.2 ± 1.6/258.4 ± 172.1	6.1 (3.9–8.3)/257 (65.2–456)
HbA1c, %	8	5.8	7.1	7.1	6.5	4.9	5.6	12.2	5.4	7.0 ± 2.2	6.5 (5.4–12.2)
Triglycerides (≥2 mmol/L)	Present	Present	Present	Present	Present from age 18 y	Present	Present	Present	Absent	NA	NA
Serum fasting triglycerides/HDL-C, mmol/L	2/1.17	3.4/0.55	3.3/0.98	4.7/1.0	5.7/0.82	6.2/0.82	6.4/0.57	6.1/NA	0.5/0.98	4.3 ± 2.1/0.86 ± 0.22	4.7 (0.5–6.4)/0.9 (0.55–1.17)
Liver steatosis	Present	Present	NA	NA	Present	NA	NA	Present	Present	NA	NA
AST/ALT, mIU/L	18/29	50/78	45/63	22/38	24/23	19/10	30/56	171/216	36/36	46.1 ± 48.1/61 ± 61.8	30 (18–171)/38 (10–216)
PCOS or hirsutism	Hirsutism; oligomenorrhea	PCOS	Absent	NA	Mild hirsutism	NA	PCOS	Oligomenorrhea	Absent	NA	NA
Glucose and lipid lowering therapy	Metformin, insulin (>2 U/kg/d), statin	Metformin, iDPP4	Metformin, insulin (>3 U/kg/d)	Metformin, HS, GLP1R-A, insulin	Metformin, GLP1R-A, statin	None	None	Metformin, GLP1R-A, insulin	None	NA	NA
Other signs	Fatigue; unexplained recurrent vomiting	Becker nevus of the shoulder; muscle fatigability	Hypertension; myocardial infarction and rhythm disturbances	None	Severe fatigue; hypertension	None	NA	NA	NA	NA	NA
*PLIN1* variant	c.1191_1192del; p.(Val398Glyfs*166)	c.1191_1192del; p.(Val398Glyfs*166)	c.1191_1192del; p.(Val398Glyfs*166)	c.1202_1205dup; p.(Pro403Argfs*164)	c.1202_1205dup; p.(Pro403Argfs*164)	c.1202_1205dup; p.(Pro403Argfs*164)	c.1202_1205dup; p.(Pro403Argfs*164)	c.1202_1205dup; p.(Pro403Argfs*164)	c.1202_1205dup; p.(Pro403Argfs*164)	NA	NA

Patient numbers refer to those shown in Fig. 1; the nomenclature of *PLIN1* variants is based on RefSeq accession numbers NM_002666.5 and NP_002657.3.

Abbreviations: ALT, alanine transaminase; AST, aspartate transaminase; DEXA, dual energy X-ray absorptiometry; GLP1R-A, GLP1R agonist; HDL-C, high-density lipoprotein cholesterol; HS, hypoglycemic sulfonamide; iDPP4, dipeptidyl peptidase-4 inhibitors; NA, not available/not applicable; PCOS, polycystic ovary syndrome.

* Proband.

Lipoatrophy had mainly affected the trunk, limbs, and femorogluteal regions and was associated with muscular hypertrophy predominantly in the calves (Fig. 1B). Lipoatrophy could be mild, especially in young patients (patients E-III1 and C-II2). Cervicofacial fat accumulation was observed in three patients (patients E-I1, C-I2, and C-II1). Low serum leptin levels and a decrease in the total fat mass, as assessed by dual energy X-ray absorptiometry, were consistent with lipoatrophy. Abdominal subcutaneous adipose tissue, studied in patients E-I1 and E-II2, displayed disorganized fat lobules of heterogeneous size, with macrophage infiltrates, increased fibrosis, and increased vascularization, consistent with previous findings (2) (Fig. 2). Seven patients showed a facial acromegaloid appearance with enlarged hands and feet. All investigated patients had presented with hyperinsulinemia or diabetes, frequently accompanied by acanthosis nigricans. Five of the seven women had polycystic ovary syndrome and/or hirsutism and oligomenorrhea. Hypertriglyceridemia was present in all investigated adult patients. No history of acute pancreatitis was reported. All examined patients had liver steatosis. Patient L-II1 had experienced major complications, including neuropathy, hypertension, and myocardial infarction with rhythm disturbances, which required the implantation of a cardioverter defibrillator.

## Discussion

The increasing use of next generation sequencing in clinical practice highlights the need for accurate interpretation of variants. When large population exome data became available, the pathogenicity of several genes involved in Mendelian disorders was questioned (7). This issue was recently raised for *PLIN1* by Laver *et al.* (6). Because of the importance of early genetic counseling for appropriate disease management (1), the clues arguing for and against a pathogenic effect of *PLIN1* null variants should be considered carefully (Table 2).

**Table 2.** Criteria for Evaluating Pathogenicity of *PLIN1* Null Variants

**Criteria supporting evidence of pathogenicity**
Absence of FPLD4-associated *PLIN1* variants in controls (Genome Aggregation Database, Exome Aggregation Consortium databases)
Enrichment of *PLIN1* frameshift variants in cohorts of patients with FPLD compared with general population
Segregation of FPLD4-associated *PLIN1* frameshift variants with the disease in eight families including 13 informative relatives
Absence of other molecular explanations for the disease in all patients with FPLD4-associated *PLIN1* frameshift variants
Homogeneity of the clinical and biological phenotype in patients with FPLD4-associated *PLIN1* frameshift variants
Demonstration of the deleterious effect of three frameshift variants in several cellular models expressing the wild-type and mutated forms of the protein
**Criteria supporting evidence of benign effect**
Elevated frequency of *PLIN1* null variants in the general population
Absence of manifestations evocative of FPLD in several individuals carrying *PLIN1* null variants

First, the allele frequency of *PLIN1* null variants in the general population, estimated at ∼4.10^−4^ in public databases, is higher than the prevalence of all forms of FPLD, estimated at ∼3.10^−6^ (8). Even if FPLD remains underdiagnosed, this suggests that certain *PLIN1* null variants are not pathogenic. However, we observed a marked enrichment of *PLIN1* frameshift variants in patients with FPLD. The allele frequency of *PLIN1* frameshift variants was estimated at 1.9% in one of our previous studies (three heterozygotes among 78 independent patients with clinically ascertained FPLD) (2) and at ∼0.8% in the present study (four positive probands for 237 tested patients).

Strikingly, the FPLD4-associated *PLIN1* variants cosegregated with the disease within the eight families available to date [Gandotra *et al.* (2), Kozusko *et al.* (3), Chen *et al.* (4), and the present study]. If these variants were all polymorphisms, the probability to see such a segregation by chance in the 13 informative affected relatives would be extremely low [(1/2)^13^; *i.e.,* 1.10^−4^).

The pathogenicity of *PLIN1* null variants was also questioned because the patients reported by Laver *et al.* (6) did not have overt lipoatrophy. As acknowledged by the authors, it can be difficult to exclude the presence of lipodystrophy in young patients, and the lack of adipose tissue-focused examinations in large cohort studies hamper the recognition of subtle lipodystrophic morphotypes. In this regard, one study underlined that FPLD remains underdiagnosed and could affect >3% of patients investigated for metabolic syndrome (9). Accordingly, lipodystrophy was diagnosed *a posteriori* in several patients in the present study, owing to the familial investigations.

Four FPLD4-associated frameshift variants lead to the synthesis of aberrant perilipin-1 isoforms [Gandotra *et al.* (2), Kozusko *et al.* (3), and the present study]. Expression in adipocyte models of three of these mutant forms of perilipin-1 decreases the size of lipid droplets and increases basal lipolysis (2, 3, 5). All but one FPLD4-associated *PLIN1* variants [p.(Val398Glyfs*166), p.(Tyr401Leufs*165), p.(Pro403Argfs*164), p.(Leu404Alafs*158)] alter the interaction domain of perilipin-1 with ABHD5 (amino acids 380 to 427). Consistently, the p.(Val398Glyfs*166) and p.(Leu404Alafs*158) mutants fail to interact with ABHD5, leading to constitutive activation of adipocyte triglyceride lipase (5). The p.(Pro439Valfs*125) mutant, which is located in the vicinity of this binding domain, fails to inhibit basal lipolysis by an alternate mechanism (3). It would be interesting to determine the functional consequences of *PLIN1* null variants identified in the general population or in those with maturity onset diabetes of the young.

At present, the classification of genetic variants follows the guidelines of the American College of Medicals Genetics and uses a five-class score (10). According to these criteria, FPLD4-associated *PLIN1* frameshift variants that disrupt protein function should be classified as “pathogenic.” Laver *et al.* (6) suggested that *PLIN1* null variants should not be reported as causative of lipodystrophy. Our study provides a set of arguments supporting the pathogenicity of several *PLIN1* frameshift variants. It is thus important to evaluate each of these variants carefully for genetic counseling. We would recommend detailed familial investigations, adipose tissue-focused examination, and careful follow-up of metabolic evolution in patients carrying such variants.

## Additional Information

***Disclosure Summary:*** The authors have nothing to disclose.

***Data Availability:*** All data generated or analyzed during this study are included in this published article or in the data repositories listed in References.

## Abbreviations:

FPLD: familial partial lipodystrophy
FPLD4: familial partial lipodystrophy type 4
